# Using large-scale perturbations in gene network reconstruction

**DOI:** 10.1186/1471-2105-6-11

**Published:** 2005-01-19

**Authors:** Thomas MacCarthy, Andrew Pomiankowski, Robert Seymour

**Affiliations:** 1 COMPLEX, University College London, 4 Stephenson Way, London NW1 2HE, UK; 2 Department of Biology, University College London, 4 Stephenson Way, London NW1 2HE, UK; 3 Department of Mathematics, University College London, Gower Street, London WC1E 2BT, UK

## Abstract

**Background:**

Recent analysis of the yeast gene network shows that most genes have few inputs, indicating that enumerative gene reconstruction methods are both useful and computationally feasible. A simple enumerative reconstruction method based on a discrete dynamical system model is used to study how microarray experiments involving modulated global perturbations can be designed to obtain reasonably accurate reconstructions. The method is tested on artificial gene networks with biologically realistic in/out degree characteristics.

**Results:**

It was found that a relatively small number of perturbations significantly improve inference accuracy, particularly for low-order inputs of one or two genes. The perturbations themselves should alter the expression level of approximately 50–60% of the genes in the network.

**Conclusions:**

Time-series obtained from perturbations are a common form of expression data. This study illustrates how gene networks can be significantly reconstructed from such time-series while requiring only a relatively small number of calibrated perturbations, even for large networks, thus reducing experimental costs.

## Background

Recent technological advances have led to an explosive growth in high-throughput genomic and proteomic data such as DNA microarrays. The rapid growth in available data has led in turn to a need for novel quantitive methods for analysis. As a consequence of this need, the reconstruction of gene network architectures from DNA microarray expression data has become a major goal in the field of systems biology. An increased understanding of the network architectures and their respective dynamics will enable novel approaches to disease treatments by allowing us, for example, to identify drug targets *in silico *which manipulate the functional outputs of these networks. This process is expected to lead to novel classes of drug based on a network approach to cellular dynamics.

Frequently, the gene expression data itself is derived from perturbation experiments such as stress conditions, temperature shifts, and chemical treatments; for example, the widely used yeast cell-cycle datasets of Cho [1] and Spellman [2]. Although these global perturbations are carried out in order to reveal causality between genes, it is not always clear how experiments should be designed so as to reveal as much causality as possible, while both minimising costly experimentation and remaining computationally tractable.

A range of computational and mathematical techniques have been adopted in the effort to find a successful gene network reconstruction technique. Reconstruction methods often have to negotiate a tradeoff between intensive (often intractable) computations, and having to perform a large number of costly experiments. Certain progress can be achieved by making simplifications, such as imposing a limit on the number of inputs to each gene, or making steady state assumptions about the system [3,4]. Some techniques described in the literature offer efficient algorithms, but require a large number of experiments, perhaps as many as there are genes [5-7]. On the other hand, theoretical work on Boolean models has shown [8] that perhaps as few as *O*(*log*(*n*)) experiments (input/output pairs) might be required for *n *genes, but that to infer these relationships requires the use of computationally costly enumeration methods.

In this paper, we propose to explore the issue of how perturbation microarray experiments might be designed, and to suggest how such experiments might be optimised so as to maximize inference capability. Logical gene network models have previously been used to investigate gene network robustness [9], perturbation dynamics [10] and evolutionary potential [11], and form the basis of the inference method used in this study. This inference method [11] is similar to others in which networks with a minimal number of connections are reconstructed through enumeration [12,13]. Given the significant speed advantage of integer computation over floating point computation, and that most genes are expected to have few inputs (93% have between 1 and 4 [14]), the method is considered to be adequate for this investigation. In this study, exhaustive evaluation was performed up to a maximum of 4 inputs of both positive and negative sign (see Methods). Enumeration is computationally feasible on an ordinary desktop computer for medium-sized networks (*n *~ 100), and still tractable for large networks (*n *~ 1000), though this would require some parallelisation. The global perturbations themselves are simulated by changing the state of each gene at random. A perturbation intensity measure *q*, defines the probability that each gene will change state (see Methods).

## Results and discussion

### A limited number of perturbations significantly improve accuracy

**Figure 1:**
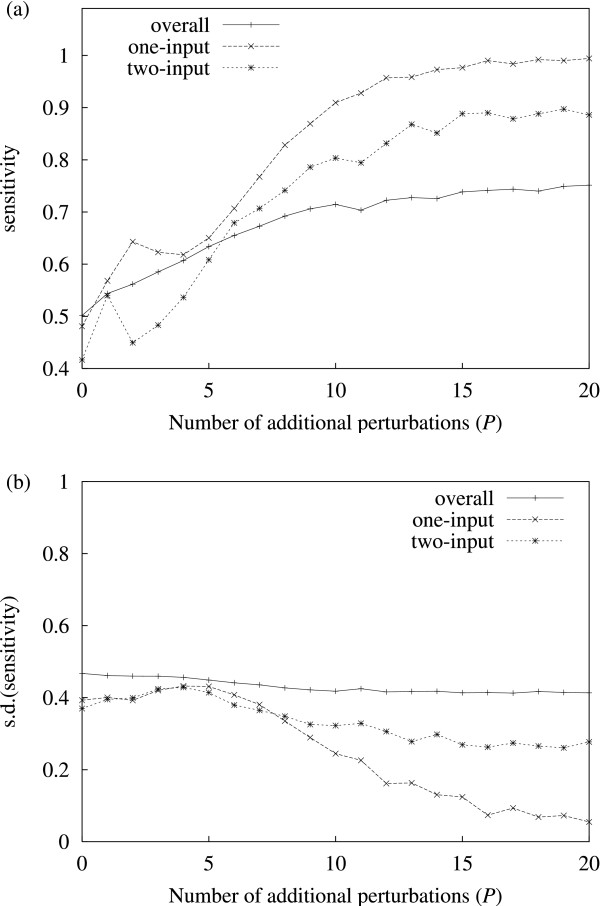
**Sensitivity vs. *P. ***(a) Sensitivity vs. number of additional perturbations used. (b) The corresponding standard deviation is shown here separately for clarity. The curves represent results for overall (i.e. all solutions) sensitivity, and specific sensitivity for (predicted) one and two-input solutions. Sensitivity is generally lower for higher order of inputs. Accuracy increases significantly with the number of additional perturbations used. The results shown are average values for 250 random networks at each data point. The remaining parameters are fixed: network size *N *= 50, perturbation intensity *q *= 0.5.

A discrete dynamical model was used to generate time series data from random networks (see Methods). To measure the effect of adding perturbations on inference ability, inference *sensitivity *(defined as true positives/true positives + false negatives, see Methods) was measured against *P*, the number of additional perturbations. Figure 1 shows the results for predicted solutions with one and two inputs, as well as overall sensitivity. The top graph in figure 1 shows that overall sensitivity is clearly enhanced by including more perturbation experiments, with lower order solutions (one and two inputs) reaching higher levels of sensitivity. The bottom graph shows the corresponding inverse relationship for the standard deviation of the sensitivity (lower for higher *P*).

It should be noted that the algorithm tends to underestimate the number of inputs a gene may have. This is to be expected in genes for which dynamics cannot be informative: for example, consider a gene *i *which has one or more negative inputs, as well as having default value OFF. Since the discrete dynamics for this gene will be the same as if it had no inputs at all (i.e. zero gene expression for *t *> 0), the presence of the inputs is impossible to infer. This underestimation effect is clear in table 1, which compares the distribution of inferred solution set sizes (|*Y*_*i*_|, see Methods) with the actual solution sizes (i.e. the indegree distribution), and shows that the method is only able to produce roughly half the number of one and two input solution sets that actually exist.

**Figure 2:**
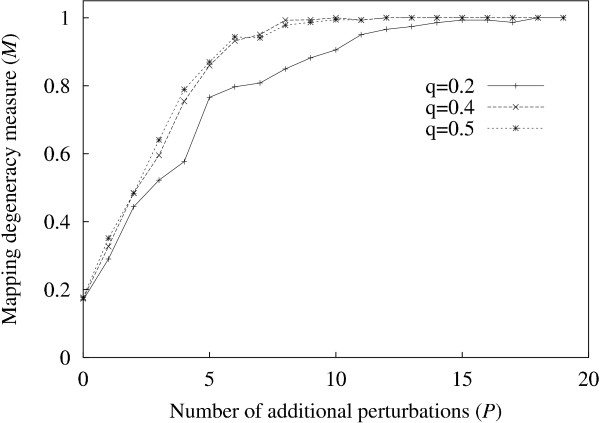
***M *vs. *P. ***M (the number of distinct "concatenated" vectors *S*_*i *_divided by N, the number of genes) increases in value, as the number of perturbations (*P*) is increased. The graph shows curves for three values of perturbation intensity *q*.

The increase in sensitivity with *P *can be explained at least partially, in the following way. Since the time series are discrete, many of the genes may have identical behaviour over time despite having different inputs (i.e. *s*_*i*_(*t*) = *s*_*j*_(*t*) for two different genes *i *and *j*). If we define a "concatenated" time series vector 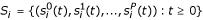 for gene *i*, and then map each gene *i *onto *S*_*i*_, we obtain a many-to-one mapping. As we increase the number of perturbations, we might expect the number of distinct time series also to increase. We define a simple measure to quantify this mapping, *M *= *n*'/*N *where *n*' is the number of distinct vectors *S*_*i*_, and *N *is the number of genes. The maximum value of *M *= 1 indicates that the mapping of genes to time series is one-to-one, whereas lower values indicate degenerate mappings. The manner in which *M *increases with the number of perturbations is shown in figure 2, and shows how the increase in M reflects the corresponding increase in sensitivity (figure 1).

### Network size and optimal perturbation intensity

The experiments described above were repeated to consider variations in two other parameters: the network size *N*, and the perturbation intensity parameter *q *(roughly, the proportion of genes whose initial expression level is changed in each perturbation experiment – see Methods).

To consider the first case, the minimum number of perturbations P* required to reach a given high accuracy criterion was measured for different values of the network size *N*. The high accuracy criterion was defined as average sensitivity = 0.95 for one-input solution sets (average sensitivity is found using a default value q = 0.5 and averaging for all the sensitivity measurements obtained from 250 random networks). To find P*, we first find the number of perturbations *P*^+^, such that average sensitivity *P*^+ ^≥ 0.95, and average sensitivity (*P*^+ ^- 1) < 0.95. If average sensitivity *P*^+ ^> 0.95, we use simple linear interpolation to find the (real) value of P* between *P*^+ ^and (*P*^+ ^- 1) for which average sensitivity = 0.95.

**Figure 3:**
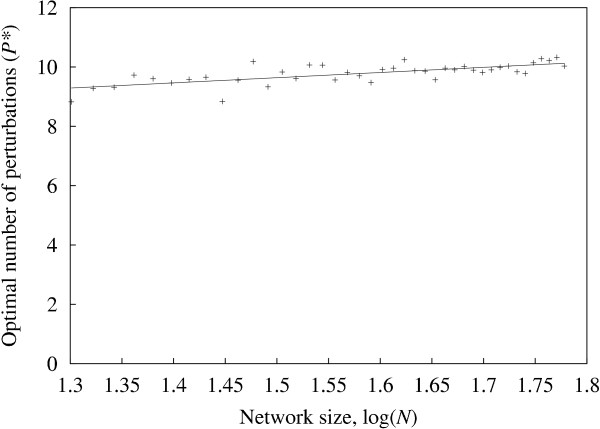
**Perturbations required for high accuracy **The minimum number of perturbations (P*) required to reach the high accuracy criterion (average sensitivity = 0.95) for different values of the network size *N*. Each point represents the average value for 250 random networks inferred. This is equivalent to finding the value of *P *for which sensitivity = 0.95 on the one-input curve of figure 1(a) for different values of *N *(figure 1(a) shows *N *= 50). A linear fit is also shown.

The resulting values for P* are shown in figure 3. Since the relationship is expected to be logarithmic [8], the plot shows *log*(*N*) against P* (logarithms used are base 10). A least squares best fit gives P* ≃ 1.75 *log*(*N*) + 7.02, which, for *N *= 1000, gives P* ≃ 12.26. In order to obtain a measure of variance for P*, we would need to calculate P*-equivalent values for many individual networks separately, then consolidate these values to obtain the relevant statistics. However, because it was only feasible to consider medium-sized networks (20 ≤ *N *≤ 70), and for any such network we often find only a small number of one-input solution sets, such statistics were found to be unreliable.

**Figure 4:**
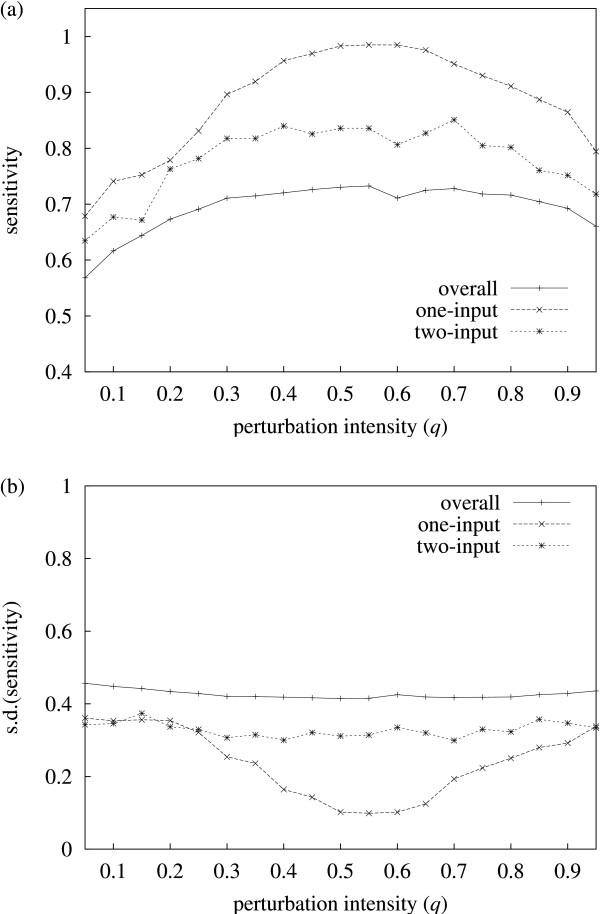
**Sensitivity vs. *q. ***(a) Average inference sensitivity vs. perturbation intensity *q*. (b) The variance (one standard deviation) is shown here separately for clarity. The results show sensitivity for (predicted) one and two-input solutions being generally higher than the overall case. The results shown are average values for 250 random networks inferred. The remaining parameters are fixed: network size *N *= 50 and *P *= 12.

The second case (varying perturbation intensity) suggests an optimal range for *q*. Figure 4a shows the inference sensitivity over a range of values for *q*, and figure 4b shows the corresponding standard deviation. Again, inference sensitivity for one-input solutions is higher than for two-input solutions, which in turn is higher than overall sensitivity. For one-input solutions, the results show a clear peak for sensitivity close to the range 0.5 <*q *< 0.6. Together with a corresponding minimisation of the standard deviation in this interval (though it still remains fairly high in absolute terms), these results suggest that perturbation intensity should be close to this range to optimise inference accuracy.

## Conclusions

A recent analysis of the yeast genetic network has shown that 93% of genes are regulated by between 1 and 4 genes [14]. This suggests that enumerative network reconstruction methods can be useful within computationally feasible limits. Experiments involving large-scale perturbations (such as temperature shifts, chemical stress) are a standard way of obtaining time-series of gene expression data [1,2]. A key result of [14] is that indegree appears to follow an exponential distribution, whereas outdegree follows a scale-free distribution, which has enabled the generation of realistic artificial gene networks used here. A logical model [11] was used to simulate the perturbed expression data. Subsequently, experimental parameters were considered in relation to inference accuracy, namely: a) number of perturbations required, *P*, and b) perturbation intensity, *q*.

The inference method itself is most useful for low order inputs, with inference accuracy maximized for predicted single input genes. More accurate methods have been proposed, though these generally require a much larger number of experiments [5,15]. Methods such as the one proposed here, which infer relationships from expression data may well be more successful when used in conjunction with other methods such as promoter analysis [16,17], or when used to drive experimental procedure [18]. Here, the results show that only a relatively small number of perturbations are necessary in order to achieve a substantial inference accuracy, even for large *N*. These relatively modest experimental requirements would presumably imply lower experimental costs. The results also suggest that the perturbations should be calibrated (by changing stress intensity, for example), so as to alter the expression levels of approximately half the genes in each experiment. Generating perturbations which alter the expression level of half the genes at random may be difficult to achieve in practice, though experiments can be designed to come as close to this goal as possible. Even in the absence of optimal perturbations, we hope the simulation approach described here will still serve as a useful tool for planning experiments.

## Methods

### Discrete dynamical model

For a system of *N *genes, the state of each gene *s*_*i *_(*i *= 1, .., *N*) is represented by the binary values 0(OFF) and 1(ON). Additionally, each gene is assigned a default ON/OFF state *θ*_*i *_∈ {0, 1}. The gene interactions are described by an (*N *× *N*) matrix *C*, composed of elements *C*_*ij *_∈ {-1, 0, +1}, representing the positive(+1), zero(0) or negative(-1) influence of gene *j *on gene *i*. State transitions are calculated as follows:


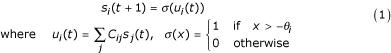


The state of the *i*th gene at the next timestep, *s*_*i*_(*t *+ 1), is therefore determined by the balance of positive versus negative inputs which are ON at the previous timestep *t*. If the balance is positive, then *u*_*i*_(*t*) > 0 and the next state will be 1(ON). Similarly, if the balance is negative, then *u*_*i*_(*t*) < 0 and the next state will be 0(OFF). If *u*_*i*_(*t*) = 0 (indicating either that there are no active input connections, or that they balance out), then the default value *θ*_*i *_determines the next state. This default value needs to be given *a priori*, and for the purpose of this study will be random.

### Network inference method

Assuming we are given the state dynamics *s*(*t*) and the default vector *θ*, the problem is to find the necessary model parameters (*C*) which will reproduce these dynamics. Specifically, a system initialised at *s*(0) should reproduce the given dynamics *s*(*t*) for *t *> 0. Note that multiple *s*(*t*) expression patterns may be defined, which will be denoted as *s*^*r*^(*t*) for *r *= 0, .., *P*, corresponding to time series with different initial states *s*^*r*^(0). Our problem is to find at least one interaction matrix that will reproduce all given dynamics *s*^*r*^(*t*). The problem of finding an appropriate matrix *C *may be broken up into *N *sub-problems, since in this system, each gene *i *may be solved independently from the others. More precisely, the inputs to gene *i *(i.e. *C*_*i*_, the *i*th row of *C*), can be found independently of the other genes. This reduces the search space from 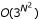 down to *O*(*N*3^*N*^). Each input *z*^*i *^to gene *i *is represented as an ordered pair (*j*, *g*), *j *∈ {1, .., *N*}, *g *∈ {± 1}, indicating an input from gene *j *of sign *g*. A solution *y*(*i*) for gene *i *is a set of *K *inputs 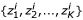 (with *y*(*i*) = *φ *if *K *= 0). For *K *inputs there are 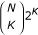 solutions to evaluate. Starting with *K *= 0 (no inputs), we progress up to a maximum of *K *= 4, exhaustively evaluating all possible solutions for each *K*. However, making a parsimony assumption, if solutions are found for some *K*_*s *_< 4, the method no longer evaluates for *K *>*K*_*s*_. Note that the method does not stop as soon as a solution is found, but evaluates all possible solutions for *K*_*s*_. The failure rate (percentage of genes for which no solution was found for *K *≤ 4) never exceeded 3% of the genes in any single network for which reconstruction was attempted.

### Global perturbations and the perturbation intensity measure

The control time series *s*^0^(*t*) is generated by setting *s*^0^(0) = *θ*. The other time series *s*^*r*^(*t*), *r *> 0 are obtained from initial conditions which are perturbations of *θ*, and correspond to standard experiments such as stress conditions, or chemical treatments. Since, experimental perturbations can usually be modulated in intensity (for example, a temperature shift), this was represented using modulated artificial perturbations. Perturbed initial states *s*^*r*^(0) were generated by randomly changing each state *s*^0^(0) with probability *q*. This means that, on average, there will be *qN *random state differences between each perturbed initial state *s*^*r*^(0), and *θ*.

### Measuring inference accuracy

Assuming one or more solutions *y*_1_(*i*), *y*_2_(*i*), ... are found for gene *i*, these are consolidated into a solution set, *Y*_*i *_= ∪_*l*_{*y*_*l*_(*i*)}. Note that some information about the solutions has been lost using this approach. For example, a solution set 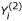 obtained from a single two-input (*K *= 2) solution: 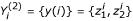, may be equal to another solution set 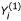 resulting from two single-input (*K *= 1) solutions: 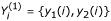 with 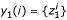 and 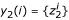.

However, this consolidation is convenient in that the solution set is easily compared with the known network structures using standard accuracy measures such as *sensitivity *and *specificity*. Here, accuracy was measured using *sensitivity*, defined as true positives / (true positives + false negatives). The relatively large number of true negatives, makes *specificity*, defined as true negatives / (true negatives + false positives), an uninformative statistic. Here, *true positives *are members of the solution set *Y*_*i *_which are also true inputs (since the networks will be generated artificially, true inputs are known), and *false negatives *are those true inputs which are not members of the solution set *Y*_*i*_.

Accuracy statistics were gathered from inferences performed on a large number of medium-sized random networks (20 ≤ *N *≤ 70). Inferences on *R *random networks (each with *N *genes), will produce approximately *RN sensitivity *measurements (slightly fewer due to the nonzero failure rate).

### Artificial gene network generation

It appears to be the case in gene networks that indegree follows an exponential distribution, whereas outdegree appears to follow a scale-free distribution. More specifically, for the yeast network, the probability distribution for indegree *k *follows *p*_*k *_~ *C*_*in*_*e*^-*βk *^with *β *~ 0.45, whereas the distribution for outdegree follows *p*_*k *_~ *C*_*out*_*k*^-*τ*^, with *τ *~ 1 (*C*_*in*_, *C*_*out *_constants) [14]. Here, artificial gene networks [19] were created using the algorithm for generating directed graphs with arbitrary in/out degree distributions described in [20]. The exponential probability distribution for indegree *k *is given by:

*p*_*k *_= (1 - *e*^-*β*^)*e*^-*βk*^,

where *β *= 0.45 is a constant. Similarly, the power law distribution (including an exponential cutoff term which is both biologically realistic and necessary analytically when *τ *< 2 [20]) for outdegree *k *is described by:

*p*_*k *_= *Ck*^-*τ *^*e*^-*γk*^,

where *C*, *γ*, and *τ *= 1 are constants. Since the algorithm begins by generating in/out-degree pairs for each node, we require equal means for both indegree (<*k*_*in *_>) and outdegree (<*k*_*out *_>). Following [20], we obtain expressions for the mean in/out degree:


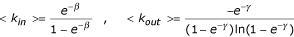


**Figure 5:**
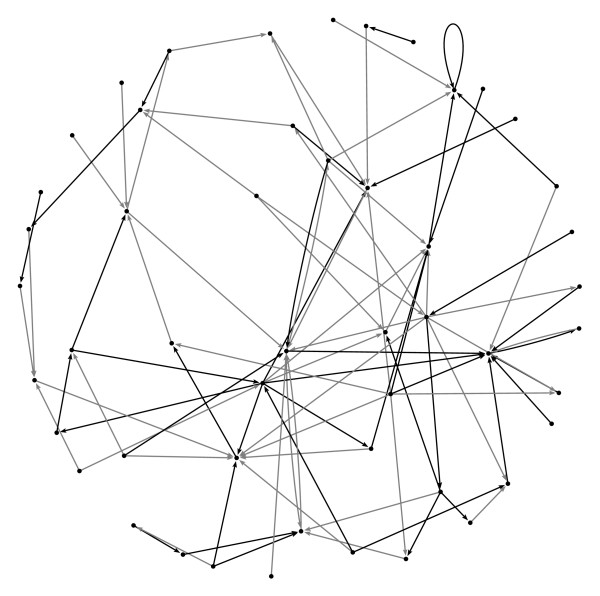
**Example network. **Example of an artificial gene network with *N *= 50. Positive interactions are shown in black, negative interactions in grey. Note the autoregulatory interaction on the upper right hand side. This diagram was generated using Pajek .

Since *β *is given, we obtain a value <*k*_*in *_> = 1.76, and fit the free parameter *γ *= 0.436 to obtain <*k*_*out *_> = <*k*_*in *_> Since the resulting networks are unweighted, non-zero weights (*C*_*ij *_∈ {-1, +1}) are assigned at random with probability 0.5, as in [19]. It should be noted that autoregulatory interactions can be (and indeed were) generated, and that these present no particular problem for the inference method. An example of a network which was used in the analysis is shown in figure 5.

## Authors' contributions

TM devised and implemented the experiments and drafted the manuscript. RS and AP supervised the project. All authors read and approved the final manuscript.

## Figures and Tables

**Table 1:** Solution set sizes Distribution for the inferred solution set sizes, compared to the distribution of indegree in the actual network for the simulations. These statistics were produced from 250 random networks run using the following parameter values: *N *= 50, *P *= 12, and *q *= 0.5. The table illustrates how the algorithm overestimates the number of solutions with zero inputs.

|*Y*_*i*_|	0	1	2	3	≥ 4
inferred	0.57	0.12	0.07	0.05	0.19
actual	0.37	0.24	0.15	0.10	0.14
